# Transformation to learning from a distance

**DOI:** 10.15694/mep.2020.000076.1

**Published:** 2020-04-23

**Authors:** David Taylor, Janet Grant, Hossam Hamdy, Leonard Grant, Hesham Marei, Manda Venkatramana

**Affiliations:** 1 Gulf Medical University; 2 Centre for Medical Education in Context (CenMEDIC)

**Keywords:** COVID-19, open learning, distance learning, e-learning, transformation

## Abstract

This article was migrated. The article was marked as recommended.

COVID-19 is a strong disruptive force that has not only influenced our global health and economy but also has changed the way we teach, learn and communicate with our students. It has disturbed the regular education pattern and the standard practices that we adapted over many years. The challenge is beyond changing the mode of delivering instructions from face to face to online. The real challenge is in creating a culture that supports the adoption of innovative practices, which require different skills and competences from the teacher, student, mentor and administrator, and at the same time maintaining the quality of the products. In other words, changing what was exceptional to be the norm over a short period of time. This article describes our approach “Open Learning” in managing such change. Our over-riding philosophy is about ensuring that students have high quality resources, and the enthusiasm and learning skills to benefit from them. At the same time we want to optimise the use of the available online applications and learning management system so that their use is within the capability of our faculty. This paper describes the evolution of our approach and the principles upon which it has been based. Our experiences over the past few months will transform the educational experience of our students over the years to come.

## Introduction

This paper is in response to the dramatic international changes in the educational landscape that have occurred since the detection of COVID-19. This strain of coronavirus has, over the course of a few months, provided us with a pandemic, and caused most nation states to adopt draconian measures to stem its progress.

In response to the threat of infection, the United Arab Emirates Ministry of Education, like many other countries ( Wikipaedia contributors, 2020), closed all institutions of higher education to students for an initial period of one month from 9 ^th^ March 2020. The Gulf Medical University, like many others, is responsible for training the health professionals who will be essential for the health and safety of the population now and in the years to come. It is therefore not an option to stop all forms of educational and clinical activity.

In philosophical terms, the Corona virus is an “Event”, which according to Alain Badiou *“has the power to rupture standard practices and approved knowledge, disclosing a heightened ability to think and act.”* ( Badiou, 2007).

We recognised that we needed to use the disruption provided by COVID-19 to develop and enhance the teaching we have normally provided, to be relevant to situations where students can no longer be together, physically on campus. It is not the first time that distance learning has been suggested as a response to such challenges ( Grant, 2001). We agree with Goh and Sandars (2020) that meeting the current challenges could, ultimately, lead to a better learning environment in the future.

At GMU, like many institutions, we opted to accept the challenge presented by the response to coronavirus and accelerate our transformation to open learning in all of our programmes. Our approach to accelerating open learning within a distance learning framework, can be seen as an Agile approach ( Beck *et al.*, 2001). The Agile framework has its history in software development where changes are delivered on a very short timescale. Working processes are favoured over comprehensive documentation and responding to change is favoured over following a plan.

Distance learning can be defined as:

Individual study of specially prepared learning materials, usually print and sometimes e-learning, supplemented by integrated learning resources, other learning experiences, including face-to-face teaching and practical experience, feedback on learning and student support. ( Grant, 2008)

Fortunately, distance learning bears a close relationship to well-organised campus-based learning:

Distance learning is itself a form of blended learning, using a variety of co-ordinated and planned modalities and methods to deliver the curriculum and enable students to learn effectively. ( Grant, 2015)

The identities of distance learning and on-campus learning are becoming blurred:

On-campus learning increasingly involves less face-to-face interaction with a teacher, while distance learning designers do their very best to ensure that distributed students have both direct and technology-mediated contact with both teachers and other students. Distance learning designers take the idea of a learning community very seriously. ( Lea and Nicoll, 2002)

In terms of reimagining course delivery and student learning in times of crises that close university campuses, the main challenges are to ensuring that faculty are competent to:

• adapt to the new delivery method.
• redesign and reuse the available instructional materials to be delivered fully online.
• integrate both synchronous and asynchronous modes of online delivery in a way that enhances students’ higher cognitive skills.
• transfer all the students’ support and mentorship activities from fully face to face to a fully online platform.

The extant use of technology to support on-campus courses, enables this conversion ( Hamadan Bin Mohammed Smart University, 2020; Lau, Yang and Dasgupta, 2020; Open University, 2020; Ross, 2020; Sanger, 2020). The methods and resources that are chosen, will be a function of the local context.

Our approach is near to that which Derek Rowntree ( Rowntree, 1977) originally called ‘materials-based learning’ and others have called ‘resource-based learning’. Based on the blended use of a variety of learning strategies, resource-based learning ‘should be based on the application of a range of instructional design principles to the development of learning materials’. In a world of abundance, the emphasis is less on the development of specific learning materials than on the selection, aggregation and interpretation of existing materials ( Ryan *et al.*, 2000).

Here we describe and explain the principles and path we have taken at Gulf Medical University.

## Principles guiding our approach

In general terms, the same cognitive theories of learning will apply regardless of delivery method or learning modality. But there are differences in:

• The techniques that are required at a distance to ensure that students fully understand how to navigate between the synchronous and asynchronous modes of online delivery
• The methods used to ensure that students are engaged in learning and are doing so effectively
• The type of management that is required of the delivery of resources and the students’ experience.

Successful distance learning requires dedicated management, a close knowledge of each student’s problems and progress, and available advice to address those.

There is a large body of literature, some of it evidence-based, to guide implementation of learning at a distance.

• In terms of the process of learning, new knowledge is built, of course, on existing knowledge and understanding and on the creation of robust, well-organised and useable cognitive structures in memory ( Bartlett, 1932; Piaget, 1952; Bruner, 1966; Baddeley, 1986; von Glaserfeld, 1989). Learning often has elements of a social activity between stakeholders ( Vygotsky, 1978) where teachers, and other students, facilitate and support individuals in their learning. We have tried to replicate this.

• Feedback on learning is also essential ( Torre, Schuwirth and Van der Vleuten, 2020) and distance learning must build this in more systematically than on-campus learning might consciously do. There are a number of potential ways of achieving this effectively at a distance. These include feedback, and reflection upon feedback given and received ( Taylor and Hamdy, 2013). This can be achieved ( Grant, 2015) in many integrated ways including through in-text activities, formative tutor-marked assignments, virtual tutorials, student real and virtual groups, on-line support and discussion forums, and assessments. Each of these might represent a minor modification to existing materials and resources.

• Distance learning must engage, guide and retain the student. In opposition to current dominant ideas about self-directed learning, distance learning works best when students are absolutely clear about the pathway that they must follow. Giving students options and choices at a distance is problematical. Distance learning students can get lost. They tend not to have a consistent or predictable approach to using such options which means that the distance teacher cannot be sure about what is being learned, unless the pathway is clear and specified ( Halbert *et al.*, 2011).

• To ensure an effective student learning pathway, distance learning must be clear in its organisation and purpose, engaging and giving a sense of achievement. It must be accessible. The actual visual presentation should be attractive, and in sections to give the student a sense of progress. There must be opportunities for students constantly to use what they have learned by the provision of learning activities. The same principles apply to materials delivered under the banner of e-learning, or as downloaded notes and workbooks or video lectures or synchronous learning events such as tutorials or seminars.

## The process of change

The challenge faced by institutions during the coronavirus pandemic is that of switching existing courses to remote learning very quickly. In our case our programs were heavily reliant on lectures, practicals and small group work, supported by the provision of some resources on-line. Our postgraduate program in Health Professions Education was already based on-line, with the addition of face to face sessions held four or five times a year. We have changed all of our programs to be on-line.

We have been able to use our existing virtual learning platform, our resources, and our experience in delivering open learning in our postgraduate courses. But this has meant a change in the way that our students and staff think about what is important in education and professional development.

The main point is not where the students are located, but in helping them to use their time to learn effectively and be able to recall and use the elements for analysis, discussion and problem-solving. So, although we had already started to use the best possible on-line resources to support our face-to-face interaction, we have discussed the ways in which we can reconceptualise the role of the lecturer and give a greater emphasis to their role in developing the individual stage of learning.

Learning delivery methods will improve and build up over time - this is an incremental process, given that it has occurred halfway through the academic year. An incremental approach also allows evaluation of effect. So this is an exciting opportunity to rethink delivery methods and to be more collaborative with students. Those who are being asked to learn from the redesigned platforms will make it clear (by their engagement, absence or feedback) what needs adjustment, and what is holding their interest and allowing them to learn.

In each of our constituent colleges and programmes at GMU, we identified academic champions who had more experience than others in using technology. Their role is to lead the change and help people to learn from each other. We also learnt through our previous experiences of on-line learning and distance education, that it is crucial to have other staff who are dedicated to making the system work. There are two reasons for this. The first is to achieve a consistent approach. The second is that it means that the academics do not get bogged down in managing the technology but can focus on the business of helping students to learn their subject.

Bates’ (2019) authoritative text shows us that online learning can encompass any use of the internet in supporting learning, providing educational experiences, and delivering materials. Although several of our staff have been involved in developing and delivering on-line learning packages over many years, we provided everyone with a simple template to plan their on-line sessions, to ensure a blend of guided learning, use of that learning, and feedback.

We held workshops and documentation to refresh people’s skills at using the on-line platform. Importantly, we held an International Virtual Workshop using “GoToMeeting”, which allowed for presentations and discussions between our Faculty, our local experts and our close partners in CenMEDIC who help deliver our Master’s Program in Health Professions Education.

## Open learning

Although there are many terms for our developing approach to education, rather than emphasise the delivery platform, we now talk about “Open Learning” in which the over-riding philosophy is about ensuring that students have high quality resources, and the enthusiasm and learning skills to benefit from them. There are three elements to this: resources, skills development and support.

Resources

Although open learning is almost invariably mediated via the web, the materials themselves should be varied and include downloadable written resources, as well as technology-based resources. Research findings consistently show that learners prefer print to other modalities ( Mizrachi, 2014).

Reasons for preferring print include:

• tactile aspects of holding, flipping and thumbing through a printed work;
• linear progression as opposed to vertical scrolling;
• better memory cues on printed pages;
• greater inclination to highlight and annotate their printed readings,
• less eyestrain and fatigue ( Mizrachi, 2014, p. 7440)

The delivery of resource materials asynchronously, where people do not all need to be on-line at the same time, is much more likely to be successful than aiming for “live” delivery. This is a function of the interface between people, their timetables and commitments, their access to technology, and also of the connectivity, bandwidth and even power supply issues that we face in some parts of the world.

The resources themselves must be mapped on to the curriculum and its timetable, and laid out in a coherent flow, just as it would be in on-campus teaching:

‘Distance learning must be seen as a whole system which integrates a wide variety of elements for curriculum coverage. These might include print, use of resources, practical classes, technology-based methods, face-to-face learning, individual and group work and many others. once the learning experiences are planned, these should be presented to students as a timetable or flow diagram, so that they are clear about what is planned for them and how the elements relate to the curriculum.’ ( Grant, 2015)

Students at a distance can get lost. So, if you move towards an on-line delivery of course material it is important to develop a clear and simple pathway to help staff and students navigate through the curriculum. A “student guide” and a “tutor guide” should be provided to help each navigate the flow and know what is expected of them. In GMU, that carefully organised flow consists of:

• Program handbooks
• Module/Unit handbooks
• Online access to resources through our VLE
  • Pre-prepared lectures and podcasts
  • Curated reference lists
  • Key resources
  • Self-assessment quizzes
• Online access to the library
• Online access to small group discussions
• Online access to team-based and problem-based learning classes
• Email support and access to other support services

We also provide discussion boards and blogs on-line for students to ask questions, clarify their ideas and receive feedback on their skills in organising the material in their own minds.

Alongside the material that needs to be understood, we also provide formative assessments, on which we try to give high value, timely, feedback. This is important, because it helps students to know how well they are understanding the material and provides encouragement to progress further. Snyder ( Snyder, 2000) discusses the way that a student’s hope for the future derives from the way in which they feel they can navigate through their program of study, and their motivation to use that pathway. Through targeted, timely and helpful feedback we can guide them to success.

The online mediated face to face element

Face-to-face teaching is a common element in distance learning, open learning and resource-based learning. It enables almost any subject and discipline to be taught at a distance, including clinical medicine ( Grant, 2008). It is important not only for teachers to ensure that they continue to know their students, but also for students to rehearse a different type of use of knowledge. Social distancing and social isolation measures mean that this is now mediated on-line.

Lectures, of course, can be migrated on-line although they need to be constructed in such a way that they can be paused for reflection and consideration, and learning activities should be provided for students to undertake as part of their listening. Webinars and on-line conferences can help groups of people discuss the issues, although they need careful preparation and planning, and access to technical support. The familiar “flipped classroom” approach to learning ( Hew and Lo, 2018) is used to help students prepare for webinars, which really should not become monologues from the teacher! Small group work and tutorials with relatively small numbers can be provided with online conference software (Zoom®, Skype®, GoToMeeting ® and others), but some regions do not permit the use of some software packages, so courses like our Master’s programs, with international participants, have to negotiate the platform to be used carefully. Our MHPE uses its own custom-made platform ( https://gmu.ac.ae/college-medicine/joint-masters-in-health-professions-education/).

Open learning can provide, and even improve on, any learning process that face-to-face learning offers. There is a literature in many fields that demonstrates this in relation to, for example, problem-based learning (PBL) (Tomaz, van der Molen and Mamede, 2013). At GMU we conduct PBL sessions online using one of the commercial systems (GoToMeeting®) while maintaining our usual PBL process. We are already replacing some of the more usual paper-based cases by introducing “virtual patient learning”, where students interact (on-line) with a pre-recorded patient describing their case ( Hamdy *et al.*, 2017; Hamdy, 2018).

Student support

Student support is required in open learning, as it is in any learning approach, but support to enable them to navigate the process may especially be required. This has been a concern since distance learning was first designed and it still is ( Tait, 1995; Zawacki-Richter, 2008). Student support not only derives from the careful design of open learning resources, but also from their management. A course manager who tracks the engagement of students, offers support and problem solving, and an academic leader who is always available via the discussion board, email or in person, are essential to students’ confidence in the process.

There is also a crucial role for technology in providing personal support. We provide support for individual students through regular one-to-one meetings on-line or by telephone, but by far the commonest form of support that is provided by and with students is on social media. The current software of choice seems to be WhatsApp® or, where that is blocked, WeChat®, but everyone reading this will realise that our students’ preferences change rapidly. The important thing is not to constrict people into a chosen pathway, but to help them use what they find most congenial.

If, when moving to remote learning, we find it hard to imagine teaching without gathering students together in a room, we can use this opportunity to consider students who have been already excluded from the classroom, for example, those with caring responsibilities or employment that means they cannot attend a face-to-face course regularly, students with physical access needs that have not been met or students who cannot use the materials in the form in which they have been provided. When moving to a remote learning framework, we can use the opportunity to make learning accessible to more students and to rethink attendance and assessment policies.

## Managing open learning

From this, it will be clear that ‘management systems that track course development and implementation, student progress and teacher activity are fundamental to success in distance learning’ ( Grant and Zachariah, 2017). Open learning requires attentive technical and interpersonal management. Regulatory authorities for distance learning set out very clearly exactly what should be managed into place to support students and ensure the smooth flow of learning ( Quality Assurance Agency for Higher Education, 2018).

Distance learning requires management and administration. Once a distance learning course starts, there are deadlines to meet, assessments to record, materials to present, students to track, communications to manage, problems to solve, records to keep.

Distance learning does not run itself. ( Grant, 2015)

## Conclusion

The COVID-19 pandemic was a disruptive event which has challenged and changed the way in which we think about and implement health professions education. We have described how we transformed from a relatively traditional medical university into one where all delivery, resources and support is delivered from a distance. To make this happen, an institution needs to be agile, identify key champions for change, and invest in the technical resources and personnel to make it possible.

We have found the process of change to be both stimulating and positive. Colleagues spend far more time thinking about how to help their students understand what is important. This pushes for even greater clarity about things that really matter. It is clear to us now that the future of health professions education, is going to be different. The challenge will be to ensure that the best of the lessons that we are learning now will survive into the future.

## Take Home Messages

• The disruptive force of the COVID-19 pandemic required a rapid shift to online delivery of resources, discussion and student support
• The process of change itself needs careful planning
• The changes needed are as much about culture as the use of hardware
• As far as possible use existing resources and familiar platforms
• Keep it as simple as possible
• Change is easier and more consistent if there are local champions who are active teachers
• Distance learning does not run itself; we need dedicated technical personnel to support and manage the process.
• Training students and staff in the new learning environment is crucial

## Notes On Contributors

**David Taylor** MA MEd PhD EdD is Professor of Medical Education and Physiology at Gulf Medical University. He has been involved in designing and delivering open learning courses since 1985, mainly at Master’s and Doctoral level, and is currently director of the Master of Health Professions Education program at GMU. He is Fellow of AMEE.

ORCiD: https://orcid.org/0000-0002-3296-2963

**Hossam Hamdy** MBCHB, FRCSEd, PhD, is Professor of Pediatric Surgery & Medical Education and Chancellor, Gulf Medical University, Ajman, United Arab Emirates. He has been involved in the development of medical schools and the delivery of medical education over many years.

ORCiD: https://orcid.org/0000-0001-6096-6139

**Janet Grant** MSc, PhD is an educational psychologist and Emerita Professor of Education in Medicine at The Open University. She worked in that distance learning institution for 35 years and collaborated in the design of a completely distance learning medical school. She has helped develop distance learning courses in India and Bangladesh and is co-Director of the FAIMER-Keele University MHPE in Accreditation and Assessment which has students from more than 40 countries. She leads on the first, distance learning, year of the GMU MHPE. She is Academic Director of the UK Centre for Medical Education in Context (CenMEDIC).

ORCiD: https://orcid.org/0000-0002-7643-4417

**Leonard Grant** MChem (Oxon) MSc LLB studied chemistry, science communication and law and is now the Course Manager and Website Designer for all the FAIMER/CenMEDIC MHPE. He takes a special interest in access and inclusion and leads on special educational needs and flexible working initiatives. He has been involved in education at a variety of levels since 2008 and is researching in decolonising the medical education curriculum.

**Hesham Marei** MSc, DDS (OMFS), PhD (Med Edu.) is a Professor of Oral and Maxillofacial Surgery at Gulf Medical University. Dr. Marei has completed his clinical oral and maxillofacial surgery training at Cardiff Dental School, UK and obtained his Fellowship in Dental Surgery in 2006 from the Royal College of Surgeons in London. Dr. Marei obtained his Master’s Degree in Medical Education in 2012 and a PhD in the Health Professions Education in 2018 at Maastricht University, the Netherlands.

ORCiD: https://orcid.org/0000-0002-5967-6473

**Prof. Manda Venkatramana**, MS, FRCSEd, FRCS(Glasg) is a Clinical Professor of Surgery and Consultant Surgeon at Thumbay Hospital. He is currently the Vice Chancellor Academics and Dean, College of Medicine at Gulf Medical University, Ajman, UAE. Prof. Manda has more than 30 years of teaching and clinical experience as a General Surgeon.

ORCiD: https://orcid.org/0000-0003-4724-3395

## Declarations

The author has declared that there are no conflicts of interest.

## Ethics Statement

Ethical permission was not required as our institution does not require approval for discussions of strategy or quality assurance projects.

## External Funding

This article has not had any External Funding
