# Supplementary material for: Transformation to learning from a distance
Source: MedEdPublish (2016). 2020 Apr 23;9:76. [Version 1] doi: 10.15694/mep.2020.000076.1 (PMC10697466; doi:10.15694/mep.2020.000076.1)
Supplement: Peer review history

# Reviewer report: Reviewer response for version 1

**DOI:** 10.21956/mep.20044.r30697

**Published:** 2020-04-29

**Authors:** Judy McKimm

**Affiliations:** 1 Swansea University

This review has been migrated. The reviewer awarded 5 stars out of 5 Thank you for writing this timely article which is very well referenced, with a lot of literature that people might not be aware of. I like the balance of practical steps and tips plus aspects of cultural and organisational change. This is a good addition to the current series and draws on the authors' extensive open learning experience. In these current times, many who are unused to a remote learning approach are having to rapidly learn on the job and any papers like this which provide useful guidance are very welcome. I would recommend this paper to those who are adapting their programmes and also maybe to learners, to help them understand some of the issues their teachers are struggling with.

Reviewer Expertise:

NA

No decision status is available

# Reviewer report: Reviewer response for version 1

**DOI:** 10.21956/mep.20044.r30700

**Published:** 2020-04-29

**Authors:** Samar Ahmed

**Affiliations:** 1 Ain Shams University Faculty of Medicine

This review has been migrated. The reviewer awarded 5 stars out of 5 I am commending this work not as a reviewer but as an end user. I think the presence of a well written reflection piece that captures Existing experience like that of Janet and other reviewers during this transformative time is very important. While we are all struggling to make this transition as less chaotic as possible it serves a great purpose to have an article mention principals guiding an approach. I think this is genius. We look at articles and their utility from the angle of whether or not the message in it is replicable and useful to readership. The basic idea that f replicability in my view is a detailed description of purpose. I believe this is generosity on the side of the authors who did not just give us a detailed description of their approach but instead helped us understand principals that in my view are the best thing to be transferred out of an educational experience. This is because principals can be manifested differently in each if our settings yet they remain solid. I think this article speaks to the “smart educator” the way I would like to be addressed

Reviewer Expertise:

NA

No decision status is available

# Reviewer report: Reviewer response for version 1

**DOI:** 10.21956/mep.20044.r30702

**Published:** 2020-04-27

**Authors:** Da-ya YANG

**Affiliations:** 1 First Affiliated Hospital of Sun Yat-Sen University

This review has been migrated. The reviewer awarded 5 stars out of 5 This paper offers practical recommendations for the management of distance learning in the midst of the COVID-19 pandemic. As a beneficiary of the programme mentioned in the article, I can personally attest to the approaches of ensuring accessible resources, online-mediated interactions, and strong support for students being instrumental in the facilitation of learning. And among the take-home messages, "The changes needed are as much about culture as the use of hardware", "As far as possible use existing resources and familiar platforms", "Keep it as simple as possible", "Change is easier and more consistent if there are local champions who are active teachers" were helpful advice for healthcare institutions trying to capitalize what they have and move forward in medical education in the coronavirus outbreak. Overall, in my opinion, this article is a must-read for all who are involved in managing distance learning programmes during turbulent times like this.

Reviewer Expertise:

NA

No decision status is available

# Reviewer report: Reviewer response for version 1

**DOI:** 10.21956/mep.20044.r30696

**Published:** 2020-04-27

**Authors:** Fan Lian

**Affiliations:** 1 the first affiliated hospital of Sun Yat-sen University

This review has been migrated. The reviewer awarded 5 stars out of 5 This paper articulates a constructive set of ideas about distance learning, which is timely and enlightening. We face a lot of challenges, but surely online teaching and learning provides opportunities to study without the limit of time and space. We have been working on this issue as well.And this paper has so many things that we are interested in.

Reviewer Expertise:

NA

No decision status is available

# Reviewer report: Reviewer response for version 1

**DOI:** 10.21956/mep.20044.r30701

**Published:** 2020-04-25

**Authors:** P Ravi Shankar

**Affiliations:** 1 American International Medical University

This review has been migrated. The reviewer awarded 5 stars out of 5 This is a very good addition to the series of articles in MedEdPublish on teaching-learning and assessment during the current pandemic. One characteristic feature of the current situation is that the apple cart has been suddenly toppled without any prior warning. So schools have to scramble together to put in place a system of online learning. I have been looking at this issue carefully over the last five weeks and I feel the ‘easiest’ component to put together may be online teaching. Many schools have simply created or rather converted their lectures slides to be delivered online. This of course, is not to be recommended. The other components to put in place are a system of assessment, system of student support and counselling, well-set mile posts, and an efficient learning or I would put it a student management system (which brings together admissions, student tracking, human resources, finance, curriculum, assessment and learning). Putting in the components like student support and assessment and linking them to competencies will be challenging. I always remember Prof Janet Grant telling us way back in 2007 about the proposal for an online medical school which the Open University had submitted to the General Medical Council. If I remember correctly she said all the components were in place but the council had its reservations. The pandemic is a challenging time and most of us are staying cooped up in our homes and under a lot of physical and mental stress. Providing proper counselling and support is important. This is true not only for students who have received most attention but also for faculty members and others. There are a lot of behind-the-scenes people who are vital for an online system to run smoothly. Educational technologists, e-learning support specialists, online support and administrative staff which many universities do not have at present. Gulf Medical University was fortunate in having a forward looking academic leadership, good technical resources, and facilities and being located in a high-income country. Another challenge I definitely foresee in many parts of the world will come from universities and councils. A form of ‘blended’ learning in my opinion is the most likely way forward. We have to educate doctors and other health care professionals who can support and protect human lives. The challenge will be less with regard to delivery but more in the areas of student support, interaction and assessment if these have to be shifted online for prolonged periods of time. The article will be of broad interest.

Reviewer Expertise:

NA

No decision status is available

# Reviewer report: Reviewer response for version 1

**DOI:** 10.21956/mep.20044.r30699

**Published:** 2020-04-25

**Authors:** John Cookson

**Affiliations:** 1 University of Worcester

This review has been migrated. The reviewer awarded 5 stars out of 5 A very topical paper in an area where most of us need some guidance

Reviewer Expertise:

NA

No decision status is available

# Reviewer report: Reviewer response for version 1

**DOI:** 10.21956/mep.20044.r30695

**Published:** 2020-04-23

**Authors:** Felix Silwimba

**Affiliations:** 1 University of Lusaka

This review has been migrated. The reviewer awarded 5 stars out of 5 this is a very informative article.brings out some of the challenges of covid-19 and the sudden switch to distance learning mode. it is a real challenge in low come countries with challenges of internet connectivity and unreliable power supply.

Reviewer Expertise:

NA

No decision status is available

# Reviewer report: Reviewer response for version 1

**DOI:** 10.21956/mep.20044.r30698

**Published:** 2020-04-23

**Authors:** Trevor Gibbs

**Affiliations:** 1 AMEE

This review has been migrated. The reviewer awarded 5 stars out of 5 During the Covid-19 pandemic, there have been many questions asked. many faculty are looking towards quick answers, much of which is not available, simply because we are moving very fast into unchartered waters. This is frequently complicated because different contexts require different answers. We do however learn from each other and this paper is one of those we can all learn from. It has some new and interesting references and refers to theories and models that many faculty may not be appreciative of. Some areas of the paper point to a common sense approach; other areas to a changed approach to a specific need, many areas are what we can all learn from.I would recommend this paper to all who are involved in changing their programmes in the light of the pandemic

Reviewer Expertise:

NA

No decision status is available
